# Detection, identification and quantification of *Campylobacter jejuni*, *coli* and *lari* in food matrices all at once using multiplex qPCR

**DOI:** 10.1186/1757-4749-6-12

**Published:** 2014-05-09

**Authors:** Lucie Vondrakova, Jarmila Pazlarova, Katerina Demnerova

**Affiliations:** 1 Institute of Chemical Technology, Prague, Faculty of Food and Biochemical Technology, Department of Biochemistry and Microbiology, Technicka 5, Prague 166 28, Czech Republic

**Keywords:** Thermotolerant *Campylobacter* spp, Multiplex qPCR, Quantification, MIQE

## Abstract

**Background:**

Thermotolerant *Campylobacter jejuni*, *coli* and *lari* are recognized as leading food-borne pathogens causing an acute bacterial enteritis worldwide. Due to narrow spectrum of their biochemical activity, it is very complicated to distinguish between individual species. For reliable risk assessment, proper incidence evaluation or swift sample analysis regarding individual species, a demand for simple and rapid method for their distinguishing is reasonable. In this study, we evaluated a reliable and simple approach for their simultaneous detection, species identification and quantification using multiplex qPCR.

**Results:**

Species specific primers and hydrolysis probes are directed to hippuricase gene of *C. jejuni*, serine hydroxymethyltransferase gene of *C. coli* and peptidase T gene of *C. lari*. Efficiencies of reactions were 90.85% for *C. jejuni*, 96.97% for *C. coli* and 92.89% for *C. lari*. At 95.00% confidence level and when cut off is set to 38 cycles, limits of detection are in all cases under 10 genome copies per reaction which is very appreciated since it is known that infectious doses are very low.

**Conclusions:**

Proposed assay was positively validated on different food matrices (chicken wing rinses, chicken juice and homogenized fried chicken strips). No inhibition of PCR reaction occurred. Assay was evaluated in accordance with MIQE handbook.

## Background

Alimentary infections caused by various food-borne pathogens generally pose a threat to public health which has to be determined and, if possible, prevented or eliminated.

Thermotolerant bacteria belonging to *Campylobacter* genus (especially *C. jejuni*, *C. coli*, *C. lari* and marginally *C. upsaliensis*) are recognised as leading human food-borne pathogens causing an acute gastrointestinal disease called campylobacteriosis. Since 2007, the incidence of campylobacteriosis in Czech Republic is significantly higher than incidence of similar well known disease salmonellosis [1]. However, this trend is also apparent in all developed countries worldwide [2].

Digestive tracts of domesticated animals farmed for meat (especially poultry, pigs, cattle and sheep) and wild warm-blooded animals are significant reservoirs of thermotolerant *Campylobacters*. However, many other sources are also known (e.g. sewage, both drinking and environmental water, raw milk, pets, various kinds of seafood, insects etc.). From all these sources they are able to spread into a food chain or to an immediate proximity of human beings and can cause the infection [3-6]. Leaving aside typical clinical symptoms [3-5,7], another significant problem is the possibility of developing various post-infectious complications e.g. reactive arthritis, urticaria or erythema nodosum. The most serious sequel is the Guillain-Barré syndrome (GBS) which manifests as an acute polyneuropathy affecting peripheral nervous system leading to a typical ascending paralysis. Especially infection caused by *C. jejuni* is a common trigger of this disease and it is estimated that its infection proceeded to about 30% of all GBS cases [3,4,8].

Although normative methods for detection and enumeration of *Campylobacter* spp. e.g. [9] show relatively high degree of specificity, their main disadvantages are apparent. These procedures are based on selective enrichment of desired bacteria, which consequently does not enable their quantification in original sample, followed by their isolation from background microflora, biochemical characterization and phenotypic or serotype identification. Considering *Campylobacters*’ special requirements for optimal growth, the detection according to standardised methods may take up to 7–10 days. Another problem appears when identification of individual species is required. This is caused mainly because of their relatively narrow spectrum of biochemical reactivity. However, also increasing numbers of nalidixic acid resistant *C. jejuni* and *C. coli* strains, or *C. jejuni* strains which are not able to hydrolyse hippuric acid under laboratory conditions (some of the biochemical tests used for species differentiation) make this situation more complicated as well [3,10-14]. Another issue linked with classical microbiological methods, which should be mentioned, is their inability to detect viable but non-culturable bacteria (VBNC). Especially *C. jejuni* is known for its capability to enter this state when stressed, starved or physically damaged. Since this phenomenon has not yet been properly explored, it is assumed that such bacteria may be able to regenerate and therefore become infectious again [15,16].

With a regard to already mentioned problems it is evident that an availability of reliable alternatives for rapid detection, identification and quantification, especially in the food and agricultural industry, is undoubtedly an issue of the day. Over the last several years, various campylobacter-focused studies implementing real-time PCR approach, which seems to be suitable to provide appropriate solution meeting all of the above requirements, have been proposed. However, only very few of them were carried out in a platform of multiplex quantitative real-time PCR (qPCR), which enables complex analysis of a given sample. Main drawbacks of proposed studies are either that they are not focused on all abovementioned main thermotolerant *Campylobacters*, and/or enable detection without species identification or quantification e.g. [17-25], or include undesired pre-enrichment step [26-28]. Also one of the most interesting studies [28] dealing with this issue suffers from several shortcomings. Briefly, *C. coli* identification is based on amplification of a gene encoding one of the subunits of a cytolethal distending toxin (*cdtA*), which is one of its virulence factors. Since *cdtA* is not housekeeping gene, is not essential and its predisposition to mutate is higher, some publications reporting data concerning its mutations and deletions in campylobacter genome have been already published and also *cdtA* negative strains are known as well [29-33]. Also the achieved detection sensitivity of the assay (about 38 genome copies per reaction), should be higher and improved by further optimization.

Moreover, during our extensive research on other assays concerned real-time PCR released after 2009, we surprisingly found no publication which would be written in accordance with so called MIQE handbook (Minimum information for publication of quantitative real-time PCR experiments), which we consider unfortunate. MIQE is very detailed set of guidelines describing the minimum information which are necessary and should be provided every time when qPCR experiments are evaluated [34-37]. Although MIQE is not obligatory, there is no doubt that such checklist helps to assure the quality of obtained results and for this reason the present study is written in accordance with it.

## Methods

### Bacterial strains and culture conditions

Bacterial strains used for experimental analyses in this study are listed in Table 1. Before further handling, all *Campylobacter* strains were incubated in Park and Sanders enrichment broth (HiMedia, India) for 24–48 h at 42°C under microaerobic atmosphere (5% O_2_, 10% CO_2_ and 85% N_2_; O_2_/CO_2_ incubator MCO-18, Sanyo, USA). Non-campylobacter bacterial strains were aerobically grown in BHI broth (brain heart infusion; Merck, Germany) for 24 h at 37°C.

**Table 1:** **List of experimentally included** ***Campylobacters*** **and other bacterial species**

**Species**	**Strain**	**Source**	**Real-time PCR specificity**
*C. jejuni* subsp. *jejuni*	CCM 6212	Human blood	+
*C. jejuni* subsp. *jejuni*	NCTC 11168	Human faeces	+
*C. jejuni* subsp. *jejuni*	81-176	Human faeces	+
*C. jejuni*	1 K	Chicken meat	+
*C. jejuni*	667/C4	Human clinical isolate	+
*C. jejuni*	681/C5	Human clinical isolate	+
*C. jejuni*	2517	Human clinical isolate	+
*C. jejuni*	3316	Human clinical isolate	+
*C. jejuni*	13	Wastewater treatment plant	+
*C. jejuni*	53G	Turkey breasts	+
*C. coli*	CCM 6211	Pig	+
*C. coli*	549/6	Wastewater treatment plant	+
*C. coli*	490/3	Wastewater treatment plant	+
*C. coli*	C253	Human clinical isolate	+
*C. coli*	C254	Human clinical isolate	+
*C. coli*	C226	Human clinical isolate	+
*C. coli*	2463	Human clinical isolate	+
*C. coli*	2521	Human clinical isolate	+
*C. coli*	2530	Human clinical isolate	+
*C. coli*	52	Chicken breasts	+
*C. lari*	CCM 4897	Herring gull, cloacal swab	+
*C. fetus* subsp. *fetus*	CCM 6213	Human blood	-
*C. upsaliensis*	ATCC 43954	Animal faeces	-
*Arcobacter butzleri*	2013/43	Chicken thigh	-
*Arcobacter cryaerophilus*	2012/1	Wastewater treatment plant	-
*Arcobacter skirrowii*	2013/34	Cow teat	-
*Bacillus cereus*	DBM 3035	Not found	-
*Bacillus megaterium*	CCM 2007	Not found	-
*Bacillus subtilis* subsp. *spizizenii*	CCM 1999	Not found	-
*Cronobacter sakazakii*	ATCC 29544	Child’s throat	-
*Enterobacter cloacae*	CCM 1903	Plasma	-
*Enterococcus faecalis*	CCM 4224	Urine	-
*Escherichia coli*	CCM 4517	Human faeces	-
*Escherichia coli*	485	Raw milk	-
*Listeria innocua*	CCM 4030	Cow brain	-
*Listeria ivanovii* subsp. i*vanovii*	CCM 5884	Sheep	-
*Listeria monocytogenes*	CCM 5576	Guinea pig mesenteric lymph node	-
*Listeria monocytogenes EGD*-*e*	ATCC BAA-679	Animal tissue	-
*Pseudomonas aeruginosa*	CCM 1968	Not found	-
*Pseudomonas aeruginosa*	49	Turkey meat	-
*Rhodococcus equi*	CCM 3429	Lung abscess of foal	-
*Salmonella enterica* subsp. *enterica*	CCM 7205	Animal tissue	-
*Staphylococcus aureus* subsp. *aureus*	CCM 3953	Clinical isolate	-
*Yersinia enterocolytica*	CNCTC 7252 Y41/73	Human faeces	-
*Yersinia ruckeri*	CCM 6093	Rainbow trout	-

ATCC – American Type Culture Collection.

CCM – Czech Collection of Microorganisms.

CNCTC – The Czech National Collection of Type Cultures.

DBM – Department of Biochemistry and Microbiology, ICT Prague.

NCTC - National Collection of Type Cultures.

### Design of qPCR assay

Material and methods section is only a brief extract from the very complex MIQE checklist in Additional file 1 where are provided all available details about experimental design and procedures.

#### DNA extraction

Genomic DNA from pure bacterial cultures (Table 1) was extracted by thermal lysis. Concentration and purity of the DNA were determined spectrophotometrically using NanoPhotometer™ (Implen, Germany). Only samples whose A_260_/A_280_ ratio ranged from 1.7 to 2.1 were used for further analyses. Real genome copy number was determined using the formula:Genome copies/*µ*l = (C × N_A_ × 10^− 9^)/(genome length(bp) × M_w_), where C is a measured concentration of extracted DNA (ng/μl), N_A_ is Avogadro’s constant (6.02 × 10^23^ molecule/mole) and M_w_ is molecular weight of 1 bp which is 660 Da [28].

#### In silico analyses

Primers and hydrolyses probes selection was based on previously published studies [18,28]. Although previously published, selected primers and probes (Table 2) have not been used together in one reaction and therefore it was necessary to perform additional *in silico* analyses. Their specificity was tested against 30 bacterial genomes (Table 3) using both basic nucleotide BLAST at NCBI (Basic local alignment search tool; National centre for biotechnology information) and FastPCR molecular biology software [38]. Additional more comprehensive analysis for primer pair specificity checking was conducted using Primer-BLAST tool at NCBI. As a database query “**Genome (chromosome of all organisms)**” was selected and as an organism query was selected “**bacteria (taxid: 2)**”. In order to enable proper optimization of qPCR protocol for a multiplex platform, necessary determination of primers and probe chemical characteristics was also carried out using FastPCR molecular biology software [38].

**Table 2:** PCR primers and probes in this assay

**Species**	**Target/GeneBank ID**	**Primer/probe**	**DNA sequence 5’→3’**	**Position within target**	**Amplicon (bp)**	**Reference**
*C. jejuni*	*hip*O^a^ NC_002163.1	Forward	TGCACCAGTGACTATGAATAACGA	809-832	124	
		Reverse	TCCAAAATCCTCACTTGCCATT	911-932		He et al., 2010 [28]
		Probe	JOE-TTGCAACCTCACTAGCAAAATCCACAGCT-Eclipse	836-864		
*C. coli*	*gly*A^b^ AF136494.1	Forward	CATATTGTAAAACCAAAGCTTATCGTG	271-297	133	
		Reverse	AGTCCAGCAATGTGTGCAATG	384-404		LaGier et al., 2004* [18]
		Probe	FAM-TAAGCTCCAACTTCATCCGCAATCTCTCTAAATTT- Eclipse	337-371		
*C. lari*	*pep*T^c^ NC_012039.1	Forward	TTAGATTGTTGTGAAATAGGCGAGTT	519-544	86	
		Reverse	TGAGCTGATTTGCCTATAAATTCG	581-604		He et al., 2010* [28]
		Probe	CY5-TGAAAATTGGAA**dC**G**dC**AGGTG-BHQ	551-570		

^a^hippurate hydrolase.

^b^serine hydroxymethyltransferase.

^c^peptidase T.

dC internal modification propynyl dC.

*alteration from reference study.

**Table 3:** **Bacterial strains for** ***in silico*** **specificity screen of primers and probes**

**Bacterial strain**	**NCBI genome accession number**
*Campylobacter jejuni* 81 - 176	NC_008787.1
*Campylobacter jejuni* 81116	NC_009839.1
*Campylobacter jejuni* NCTC 11168	NC_002163.1
*Campylobacter coli* JV20	AEER01000001.1
*Campylobacter lari* RM2100	NC_012039.1
*Campylobacter concisus* 13826	NC_009802.1
*Campylobacter curvus* 525.92	NC_009715.1
*Campylobacter fetus* subsp. *fetus* 82-40	NC_008599.1
*Campylobacter hominis* ATCC BAA-381	NC_009714.1
*Arcobacter butzleri* RM4018	NC_009850.1
*Arcobacter nitrofigilis* DSM 7299	NC_014166.1
*Bacillus cereus* ATCC 10987	NC_003909.8
*Bacillus subtilis* subsp. *subtilis* str. 168	NC_000964.3
*Cronobacter sakazakii* ATCC BAA-894	NC_009778.1
*Enterobacter aerogenes* KCTC 2190	NC_015663.1
*Enterobacter cloacae* subsp. *cloacae* ATCC 13047	NC_014121.1
*Escherichia coli* ATCC 8739	CP000946.1
*Escherichia coli* BW2952	NC_012759.1
*Helicobacter pylori* 26695	NC_000915.1
*Listeria monocytogenes* 08-5578	NC_013766.1
*Listeria monocytogenes* EGD-e	NC_003210.1
*Pseudomonas aeruginosa* UCBPP-PA14	NC_008463.1
*Salmonella bongori* NCTC 12419	NC_015761.1
*Salmonella enterica* subsp. *enterica* serovar Typhimurium str. LT2	NC_003197.1
*Shigella boydii* CDC 3083-94	NC_010658.1
*Shigella dysenteriae* Sd197	NC_007606.1
*Shigella flexneri* 2002017	CP001383.1
*Shigella sonnei* Ss046	NC_007384.1
*Staphylococcus aureus* subsp. *aureus* NCTC 8325	NC_007795.1
*Yersinia enterocolitica* subsp. *enterocolitica* 8081	NC_008800.1

#### Empirical primers’ specificity screen

In addition to *in silico* analyses experimental primers’ specificity verification was implemented. Horizontal agarose-gel electrophoresis and melt curve analysis utilizing SYBR Green fluorescent dye (2×Power SYBR Green PCR Master Mix, Applied Biosystems, USA) were performed. All target and non-target *Campylobacter* species listed in Table 1 were used for these analyses.

#### Singleplex qPCR

Choice of probes and primers concentrations for the first experiments was based on our research on previously published assays where the most common final concentrations were 0.40 μM and 0.20 μM for primers and probes respectively e.g. [22,24,39-42]. Before standard curves were generated, one experiment was carried out with all target and non-target *Campylobacter* species as well as with other bacterial strains (Table 1) in order to experimentally verify also specificity of probes. After the positive verification only three reference target *Campylobacter* species (*C. jejuni* CCM 6212, *C. coli* CCM 6211 and *C. lari* CCM 4897) were used for further experiments. Standard curves for singleplex qPCR platform were generated as described in Additional file 1. All samples were run in duplicates and non-template (NTC) and positive controls were included.

#### Multiplex PCR

Multiplex qPCR was evaluated in two phases. In the first phase, reaction was performed with all components (primers and probes) with DNA from one strain in a tube in order to find out whether undesirable inhibition caused by interaction between components occurs or not. Standard curves were generated and quantification cycles (Cq), y-intercepts, slopes, efficiencies (E), standard deviations (SD), correlation coefficients (R^2^) and linear ranges were determined using 7500 Software (Applied Biosystems, USA, version 2.0.5), Microsoft Office Excel 2007 (Microsoft, USA, version 2007) and GenEx software (MultiD Analyses AB, Sweden, version GenEx 5 Enterprise).

In the second phase, reaction mixture contained all chemical components as well as mixed DNA sample from all target *Campylobacter* strains. Standard curves were generated and abovementioned parameters determined using the same approach. In accordance with obtained results the concentrations of individual components and reaction conditions were optimized until satisfying values were obtained (Additional file 1).

#### Data analysis

Output data were analysed with instrument compliant 7500 Software (Applied Biosystems, USA, version 2.0.5), Microsoft Office Excel 2007 (Microsoft, USA, version 2007) and GenEx software (MultiD Analyses AB, Sweden, version GenEx 5 Enterprise).

### Assay validation on food samples

#### Sample collection and processing

Three different types of food matrices were chosen for empirical assay validation – raw chicken wings (local butchery, Prague, Czech Republic), whole frozen chicken without giblets (local hypermarket, Prague, Czech Republic) and fried chicken strips (fast food restaurant, Prague, Czech Republic). All samples were processed immediately after the purchase as described in Additional file 1, and obtained chicken wing rinses, chicken juice and homogenate from fried chicken strips were further examined.

#### Artificial contamination of food samples

Each sample was divided into four aliquots. One remained unspiked and the rest was artificially contaminated with pure culture of individual reference *Campylobacter* strain and then serially diluted in order to achieve the range of approximately 10^1^-10^5^ CFU/ml. The number of *Campylobacter* cells used for the spiking was determined on Karmali agar with *Campylobacter* selective supplement (sodium pyruvate, vancomycin, cefoperazone and cycloheximide; Oxoid, UK) using a drop plate method [43], by our qPCR as well as via genome copy number determination in pure cultures used for spiking [28].

#### DNA extraction and qPCR

DNA was isolated from 750 μl of each food sample using commercial PrepSEQ® Spin Sample Preparation Kit with Protocol (Applied Biosystems, USA) in accordance with manufacturer’s recommendations.

qPCR reaction was performed as described in Additional file 1. Briefly, standard curves were constructed using 5 μl of mixed DNA extracted from target *Campylobacters*. Used DNA concentrations ranged approximately from 10^0^ to 10^6^ genome copies (CFU equivalent) per well for *C. jejuni* and from 10^0^ to 10^5^ for *C. coli* as well as for *C. lari*. In the case of food samples 10 μl of DNA were added into reaction. Simultaneously, all food samples were examined for presence of *Campylobacters* using drop plate method [43].

## Results and discussion

### qPCR assay

For implementation of multiplex qPCR assay three species specific target genes (Table 2) were selected based on research of previously published studies e.g. [23,27,28,44-49]. Considering the very short length and high similarity [50] of campylobacters genomes (from 1.5 to 1.7 Mbp), it was necessary to carefully choose such primers and probes which interact exclusively with its target gene, do not form any secondary structures and also have similar chemical characteristics in order to allow the co-amplification of multiple targets in one tube without any competition or inhibition. Regarding obtained results, *C. lari* specific probe, previously designed by another research group [28], was additionally internally modified with propynyl at two cytosines (Eastport, Czech Republic) because of its shorter length and therefore lower melting temperature in comparison with the others. Our modification increased its melting temperature by 5°C and therefore its utilization in multiplex platform was possible.

Primers’ specificity was experimentally verified with simple horizontal agarose-gel electrophoresis and melt curve analysis performed with SYBR Green fluorescent dye. DNA from target and non-target *Campylobacter* species (Table 1) was used. As expected, only specific melting peaks of amplified products were obtained. Nonspecific amplicons of different lengths or primer-dimers did not form. Amplification of non-target DNA (*C. fetus* subsp *fetus* CCM 6213 and *C. upsaliensis* ATCC 43954) did not occur as well.

#### Singleplex qPCR

First singleplex qPCR served for specificity screen of hydrolysis probes. As a sample DNA isolated from all bacteria listed in Table 1 was used. Three different combinations of primers and probes final concentrations in reaction were tested as follows: 0.40×0.20, 0.30×0.10 and 0.20×0.05 μM respectively. There were no significant differences between Cq values, therefore for further experiments in singleplex platform the combination of concentrations 0.40×0.20 μM were used. No fluorescent signal was detected when non-target DNA was used as a sample. When specificity of probes was positively verified standard curves for target *Campylobacters* were generated. Quantification cycles and efficiencies were 18.54 and 84.56% for *C. jejuni*, 25.27 and 87.11% for *C. coli*, 16.19 and 77.93% for *C. lari* (Table 4) when 10^6^ genome copies per well used. Because of very high Cq value for *C. coli* when compared with the others, another combinations of primer and probe concentrations were tested as follows: 0.40×0.20 to 0.50, 0.50×0.20 to 0.50 and 0.80×0.80 μM. However, no significant differences were observed. Another *in silico* analysis showed one non-complementary base at 3’ end of the forward primer, which was not issue when used in original study [18] where only duplex qPCR was evaluated and quantification cycles ranged between 18.80-23.00 (when 10^6^ genome copies per well used). Therefore 3’ end of original primer was amended by adding a two bases which increased a stability and specificity of annealing step (Additional file 1). Our adjustment caused significant decrease in Cq value to 15.72 (when 10^6^ genome copies per well used) and slight increase in efficiency as well (89.24%) when concentrations of primers and probes in reaction were 0.40×0.20 μM (Table 4). Due to the fact that singleplex platform was mainly performed in order to verify the functionality of the reaction, we proceeded directly to the multiplex without further optimization in order to improve obtained values.

**Table 4:** Comparison of results for singleplex qPCR and optimized multiplex qPCR with pure cultures

**Platform**	**Singleplex**				**Multiplex**			
**Strain**	**Efficiency (%)**	**Slope**	**y-intercept**	**R**^**2**^	**Efficiency (%)**	**Slope**	**y-intercept**	**R**^**2**^
*C. jejuni* CCM 6212	84.560	−3.757	41.308	0.999	90.848	−3.565	42.227	0.998
*C. coli* CCM 6211	89.235	−3.610	37.393	1.000	96.973	−3.399	40.040	0.998
*C. lari* CCM 4897	77.930	−3.996	40.234	0.993	92.888	−3.506	39.548	0.998

R^2^ correlation coefficient.

#### Multiplex qPCR

In the first experiment, all components were present in reaction mixture at the same concentration as in the singleplex, but DNA sample in each reaction originated only from individual species (not mixed sample). Considering greater number of components when multiplexing, reaction volume was increased from 25 μl to 30 μl. Results showed that there is no inhibition of the reaction caused by interaction between components. Serial dilutions of DNA were in the range of 10^0^-10^7^ genome copies (CFU equivalents) per well. Quantification cycles and efficiencies were 23.80 and 91.35% for *C. jejuni*, 23.41 and 95.23% for *C. coli* and 21.53 and 92.12% for *C. lari* when 10^5^ genome copies per well used (more details in Additional file 1).

In second phase multiplex with mixed DNA sample was evaluated. First experiment was carried out under the same conditions as the singleplex. Serial dilutions of mixed DNA were in the range of 10^0^-10^7^ genome copies of each strain per well. Although Cq values and efficiencies for *C. coli* and *C. lari* were comparable with previous multiplex results (DNA from single strain), it was unambiguous that strong inhibition of amplification occurred in the case of *C. jejuni* because of a complete disappearance of its PCR product. Therefore conventional multiplex PCR (all three pairs of primers and three probes) with end point horizontal agarose-gel electrophoresis was conducted with two possible combinations of DNA present in sample (*C. jejuni*×*C. coli*; *C. jejuni*×*C. lari*) in order to determine in which case the problem occurs. Based on results it was found that when all components are present in reaction with DNA sample mixed of *C. jejuni* and *C. lari* the amplification of *C. jejuni* target is affected and the typical PCR product does not form. Having regard to the fact that there was no inhibition due to competition for other reaction components when multiplex with DNA sample from each strain individually was performed, this indicated that there was some interaction between *C. jejuni* and *C. lari* DNA even though the trend of *C. lari* reaction was not affected at all. Considering this fact, another optimization was necessary and various concentrations of *C. jejuni* and *C. lari* specific primers and probes were tested (results not shown).

Fully optimized reaction mixture consisted of 0.80 μM *C. jejuni*, 0.40 μM *C. coli* and 0.05 μM *C. lari* primers and 0.20 μM of each probe. Eight points of ten-fold serial dilutions in the range of 10^0^-10^7^ genome copies of each strain per well were used to generate standard curves. Values of quantification cycles and efficiencies are 22.99 and 90.85% for *C. jejuni*, 20.77 and 96.97% for *C. coli*, 20.04 and 91.05% for *C. lari*, when 10^5^ genome copies per well used (Table 4). All reactions were linear over seven orders of magnitude in the range 10^1^-10^7^ with potential to cover wider range in higher orders. Detection limits of this assay were determined to be between 6.62-16.10 genome copies/well for *C. jejuni*, 5.13-6.30 genome copies/well for *C. coli* and 4.87-5.23 genome copies/well for *C. lari*. All other parameters are provided in Additional file 1.

### Food sample analyses

For empirical assay evaluation on food samples, three different food matrices which were likely to be naturally contaminated with *Campylobacter* species were examined (chicken wing rinses, chicken juice and homogenate prepared from fried chicken strips). Sample aliquots were artificially contaminated with individual target *Campylobacters*. Unspiked samples were tested for natural contamination as well. Plate counting method [43] and proposed qPCR assay were simultaneously compared.

Using qPCR, quantification of target *Campylobacters* was possible in all tested food samples even when the highest dilutions were used for spiking. Quantification by plate counting was always possible in the range of 10^3^-10^5^ CFU/ml however, for some target *Campylobacters* failed when higher dilutions were used for spiking (Table 5). Quantification of *C. jejuni* by plate counting, regardless of the sample analysed, always failed with the dilution corresponding to 10^2^ CFU/ml and higher in which case no growth on the plates was observed even after prolonged incubation for 72 hours. Therefore this concentration seems to be the detection limit for this specie when the plate counting is used. All the unspiked food samples were determined to be *Campylobacter* free by plate counting. On the contrary, the unspiked chicken juice was determined to be naturally contaminated by *C. jejuni* using qPCR (Table 5). Also one of the unspiked chicken rinses was reliably determined to be naturally contaminated by *C. coli* however, its numbers were below quantification limit. As mentioned above, there is a possibility that food samples or *Campylobacter* cultures used for spiking contained certain numbers of dead or VBNC cells, which were detected and quantified with qPCR but did not grow on plates. However, considering fact that cultures were fresh and under no stress, it is highly unlikely that number of such cells would be significant.

**Table 5:** Comparison of food sample analyses results obtained by plate counting and qPCR

	**Food sample**														
	**Wing rinse A**			**Wing rinse B**			**Wing rinse C**			**Chicken juice**			**Fried strips homogenate**		
**CFU/ml**	**10**^**1**^	**10**^**2**^	**qPCR**	**10**^**1**^	**10**^**2**^	**qPCR**	**10**^**1**^	**10**^**2**^	**qPCR**	**10**^**1**^	**10**^**2**^	**qPCR**	**10**^**1**^	**10**^**2**^	**qPCR**
*C. jejuni*	-	-	-	-	-	-	-	-	-	-	-	7.1×10^2^ ± 1.6×10^2^	-	-	-
*C. coli*	-	+	-	+	+	-	-	+	+^*^	-	+	-	+	+	-
*C. lari*	+	+	-	-	+	-	+	+	-	+	+	-	-	+	-

- negative quantification.

+ positive quantification.

*quantification was not possible, because cell numbers were below quantification limit of qPCR.

## Conclusions

In conclusion, we provided a reliable method for detection, identification and quantification of three most abundant thermotolerant *Campylobacters*. The main advantage of this approach over normative methods for their characterization is a possibility to exclude the pre-enrichment step. Exclusion of this part dramatically reduces the time required for analysis. Also the possibility to identify all three species at once is appreciated, since cases of co-contamination and co-infection with more than one *Campylobacter* specie are relatively common [51-53]. Also this publication is written in accordance with the MIQE handbook [35,36] which introduces a very good way to establish a consensus on how best to perform and interpret qPCR experiments in order to facilitate cooperation between laboratories, comparability and reproducibility of obtained results, and generally serves for higher standardization of real-time PCR experiments.

## Competing interests

Authors declare that they have no competing interests.

## Authors’ contributions

LV, JP and KD participated in the design of the study. LV performed experiments, collected and analysed data. All authors provided ideas, comments and prepared a draft manuscript and approved the final manuscript.

## Supplementary Material

Additional file 1 Supplementary text 1. Click here for file
