# Supplementary material for: Detection, identification and quantification of Campylobacter jejuni, coli and lari in food matrices all at once using multiplex qPCR
Source: Gut Pathog. 2014 May 9;6:12. doi: 10.1186/1757-4749-6-12 (PMC4108124; doi:10.1186/1757-4749-6-12)
Supplement: Additional file 1 — Supplementary text 1. [file 1757-4749-6-12-S1.docx]

**Additional file 1**

**MIQE guidelines**

Minimum Information for Publication of Quantitative Real-Time PCR Experiments

Essential information (E) and Desirable information (D)

**1. Experimental design**

**Definition of experimental and control groups (E) and number within each group (E)**

i) Twenty-three previously characterized *Campylobacter* strains were used for experimental analyses (Table 1).

ii) Twenty-two previously characterized bacterial strains other than *Campylobacter* were used for specificity screening (Table 1).

iii) 30 bacterial strains (genome sequences respectively) were used for *in silico* analyses (Table 3).

**Assay carried out by the core or investigator's lab? (D)**

Assay was carried out by investigator's lab

**2. Sample**

**Description (E)**

Pure cultures of abovementioned *Campylobacter* strains (Table 1) were included in an experimental design of *de novo* protocol. Culture conditions – Park and Sanders broth (HiMedia, India); 24-48 h; steadily; at 42°C; under microaerobic atmosphere (5% O_2_, 10% CO_2_ and 85% N_2_; O_2_/CO_2_ incubator, MCO-18, Sanyo, USA). The strains *C. jejuni* CCM 6212, *C. coli* CCM 6211 and *C. lari* CCM 4897 were further used also for food sample spiking.

Pure cultures of abovementioned non-campylobacter bacterial strains (Table 1) were included for specificity screening. Culture conditions – BHI broth (brain heart infusion; Merck, Germany); 24 h; steadily; at 37°C; aerobically.

Three chicken wings (local butchery, Prague, Czech Republic), whole frozen chicken without giblets (local hypermarket, Prague, Czech Republic) and fried chicken strips (fast food restaurant, Prague, Czech Republic) were used for validation of designed protocol when applied on different real food samples.

**Microdissection or Macrodissection (E)**

Not applied

**Processing procedure (E)**

Chicken rinse preparation:

Each wing was hand massaged in plastic bag containing 65 ml of physiological saline for 3 minutes. Afterwards, the wing was removed and 50 ml of the rinse centrifuged for 15 min at 16000 × g. The supernatant was discarded and the pellet was resuspended in 50 ml of the second rinse which was conducted under the same conditions. In order to remove larger particles from rinse fluid low-speed centrifugation was applied (5 min at 1880 × g). Supernatant was transferred to another tube and centrifuged for 15 min at 16000 × g. The supernatant was discarded and the pellet was resuspended in 50 ml of physiological saline.

Chicken juice preparation:

Commercially frozen chicken was placed into a container and left to thaw overnight at ambient temperature. About 20 ml of chicken juice was collected and centrifuged for 15 minutes at 16000 × g. The supernatant was discarded and the pellet resuspended in a 40 ml of physiological saline. In order to remove larger particles from the sample low-speed centrifugation was applied (5 min at 1880 × g). The supernatant was transferred to another tube and centrifuged for 15 min at 16000 × g. The supernatant was discarded and the pellet was resuspended in 50 ml of physiological saline.

Preparation of fried chicken strips homogenate:

Homogenate sample was made by adding 100 g of fried chicken strips to 200 ml of physiological saline into a plastic bag with a filter and mixing in a stomacher for 3 minutes. Afterwards, 50 ml of the homogenate were centrifuged for 15 min at 16000 × g. The supernatant was discarded and the pellet was resuspended in 50 ml of a physiological saline. In order to remove larger particles from the sample low-speed centrifugation was applied (5 min at 1880 × g). The supernatant was transferred to another tube and centrifuged for 15 min at 16000 × g. The supernatant was discarded and the pellet was resuspended in 50 ml of physiological saline.

**If frozen, how and how quickly? (E)**

Not applied

**If fixed, with what and how quickly? (E)**

Not applied

**Sample storage conditions and duration (E)**

Food samples were processed immediately after the purchase.

**3. Nucleic acid extraction**

**Procedure and/or instrumentation (E)**

i) Thermal lysis (protocol designed and adjusted in investigator's laboratory):

From pure bacterial cultures DNA was extracted from the volume of 1 ml. Suspension was centrifuged for 10 min at 10000 × g. The supernatant was discarded and the pellet was resuspended in 1 ml of physiological saline and then centrifuged for the second time under the same conditions. The supernatant was discarded and the pellet was resuspended in 100 µl of nuclease-free water (Promega, USA). Lysis was performed at 95°C for 20 min. Cell lysate was immediately cooled on ice, shortly vortexed and then centrifuged for 3 min at 10000 × g. Extracted DNA was present in the supernatant (if needed for further experiments, DNA was stored at -20°C)

ii) DNA extraction kit:

DNA from all food samples was isolated using commercial PrepSEQ® Spin Sample Preparation Kit with Protocol (Applied Biosystems, USA) in accordance with manufacturer's recommendations.

**Name of kit and details of any modifications (E)**

PrepSEQ® Spin Sample Preparation Kit with Protocol (Applied Biosystems, USA)

**Details of DNase or RNase treatment (E)**

Not applied

**Contamination assessment (DNA or RNA), (E)**

Not applied

**Nucleic acid quantification (E)**

DNA concentration (ng/µl) was determined spectrophotometrically using NanoPhotometer^TM^ (Implen, Germany)

**Instrument and method (E)**

NanoPhotometer^TM^ (Implen, Germany)

Performed in accordance with manufacturer's recommendations

**Purity (A_260_/A_280_), (D)**

DNA purity was determined spectrophotometrically using NanoPhotometer^TM^ (Implen, Germany)

Only samples whose A_260_/A_280_ ratio ranged from 1.7 to 2.1 were used for further analyses (indication of good DNA purity)

**RNA integrity: method/instrument (E)**

Not applied

**RIN/RQI or C_q_ of 3' and 5' transcripts (E)**

Not applied

**Inhibition testing (C_q_ dilutions, spike, or other), (E)**

Serial dilutions followed by qPCR and Cq determination were performed (details in section 7 – qPCR protocol; Complete reaction conditions)

No inhibition was observed when DNA isolated from pure cultures was used.

Inhibition occurred only when DNA from food samples (chicken rinses) was isolated by thermal lysis which was obviously because of a high level of inhibiting components. Therefore only DNA isolated by commercial PrepSEQ® Spin Sample Preparation Kit with Protocol (Applied Biosystems, USA) was further used when food samples processed.

Inhibition testing during food sample analyses:

Food samples (chicken juice and homogenate prepared from fried chicken strips) were spiked with mixed suspension of the *C. jejuni* CCM 6212, *C. coli* CCM 6211 and *C. lari* CCM 4897 (final concentration of each strain was 10^1^ CFU/ml). Simultaneously mixed pure suspension of the same final cell concentration was prepared in physiological saline. DNA was isolated from 750 µl using commercial PrepSEQ® Spin Sample Preparation Kit with Protocol (Applied Biosystems, USA). Multiplex qPCR was performed as described in section **7. qPCR protocol**. Quantification cycles were compared and no inhibition during PCR reaction was observed (Table 6).

Table 6: Results of inhibition testing during food sample analyses

| Strain | Cq mean | | | | | |
| --- | --- | --- | --- | --- | --- | --- |
|  | physiological saline | SD | chicken juice | SD | fried strips homogenate | SD |
| *C. jejuni* | 32.66 | 0.24 | -^*^ | -^*^ | 32.30 | 0.08 |
| *C. coli* | 32.44 | 1.15 | 33.70 | 0.15 | 32.77 | 0.17 |
| *C. lari* | 31.21 | 0.27 | 31.47 | 0.19 | 32.06 | 0.09 |

^*^ chicken juice was naturally contaminated with therefore inhibition testing for this specie was not possible

**4. Reverse transcription**

Not applied

**5. qPCR target information**

**Gene symbol (E)**

*hipO, glyA, pepT*

**Sequence accession number (E)**

*hipO* NC_002163.1, *glyA* AF136494.1, *pepT* NC_012039.1

**Location of amplicon (within target), (D)**

*hipO* 809-932, *glyA* 271-404, *pepT* 519-604

**Amplicon length (E)**

*hipO* 124 bp, *glyA* 133 bp, *pepT* 86 bp

***In silico* specificity screen (BLAST, and so on), (E)**

NCBI standard nucleotide BLAST (nBLAST) – specificity check of primers and probes against 30 bacterial genomes (Table 3; control group iii) in section 1 – Definition of experimental and control groups)

Additional more comprehensive *in silico* analysis for primer pair specificity checking was conducted using Primer-BLAST tool at NCBI. As a database query “**Genome (chromosome of all organisms)**” was selected and as an organism query was selected “**bacteria (taxid:2)**”.

**Secondary structure analysis of amplicon (D)**

FastPCR© molecular biology software [[1](#_ENREF_1)] – prediction of amplicons' sizes, melting temperatures, GC content and secondary structure formations

**Location of each primer by exon or intron (if applicable), (E)**

Not applied – prokaryote

**What splice variants are targeted? (E)**

Not applied – prokaryote

**6. qPCR oligonucleotides**

**Primer sequences, 5'→ 3', (E)**

*hipO* [[2](#_ENREF_2)]

F: tgcaccagtgactatgaataacga, R: tccaaaatcctcacttgccatt

*glyA* [[3](#_ENREF_3)] with alteration: 3' end modification in forward primer (this section below – Location and identity of any modifications)

F: CATATTGTAAAACCAAAGCTTATCGTG, R: agtccagcaatgtgtgcaatg

*pepT* [[2](#_ENREF_2)]

F: ttagattgttgtgaaataggcgagtt, R: tgagctgatttgcctataaattcg

**Probe sequence, 5'→ 3', (D)**

*hipO [*[*2*](#_ENREF_2)*]*

JOE-TTGCAACCTCACTAGCAAAATCCACAGCT-eclipse

*glyA* [[3](#_ENREF_3)]

FAM‑TAAGCTCCAACTTCATCCGCAATCTCTCTAAATTT- eclipse

*pepT* [[2](#_ENREF_2)]; with alteration (this section below – Location and identity of any modifications)

Cy5-TGAAAATTGGAA**dC**G**dC**AGGTG-BHQ

**Location and identity of any modifications (E)**

forward primer *glyA* altered from reference publication [[3](#_ENREF_3)] – two bases at 3**'** end

original forward primer: CATATTGTAAAACCAAAGCTTATCG**G**

altered forward primer: CATATTGTAAAACCAAAGCTTATCG**TG**

*pepT* probe altered from reference publication [[2](#_ENREF_2)] – two internal modifications: propynyl dC

original probe: TGAAAATTGGAACGCAGGTG

altered probe: TGAAAATTGGAA**dC**G**dC**AGGTG

**Manufacturer of oligonucleotides (D)**

East Port, Prague, Czech Republic

**Purification method (D)**

Primers – desalted; Probes – HPLC

**7. qPCR protocol**

**Complete reaction conditions (E)**

1. SYBR Green melt curve analysis:

For primers' specificity and secondary structures formation check

DNA samples: *C. jejuni* subsp. *jejuni* CCM 6212, *C. coli* CCM 6211, *C. lari* CCM 4897, *C. upsaliensis* ATCC 43954 and *C. fetus* subsp. *fetus* CCM 6213

Cycling parameters: from 60°C to 95°C

Reaction mixture consisted of 12.5 µl 2 × Power SYBR Green PCR Master Mix (Applied Biosystems, USA), 1 µl of each 10 µM primer (0.4 µM final), 5.5 of µl nuclease-free water (Promega, USA) and 5 µl of DNA; final volume 25 µl

1. Singleplex qPCR:

DNA samples: Genomic DNA isolated from all bacteria listed in Table 1.

Cycling parameters: 95°C for 10 min, followed by 40 cycles consisting of 95°C for 20 s and 60°C for 60 s

Each reaction mixture consisted of 12.5 µl 2 × TaqMan® Universal PCR Master Mix, No AmpErase® UNG (Applied Biosystems, USA), 1 µl of each 10 µM primer (0.4 µM final), 0.5 µl of 10 µM hydrolysis probe (0.2 µM final), 5 µl of nuclease-free water (Promega, USA) and 5 µl of DNA; final volume 25 µl

1. Optimized multiplex qPCR:

Cycling parameters: 95°C for 10 min, followed by 40 cycles consisting of 95°C for 20 s and 60°C for 60 s

Reaction mixture consisted of 15 µl 2 × TaqMan® Universal PCR Master Mix, No AmpErase® UNG (Applied Biosystems, USA), 0.24 µl of 100 µM *C. jejuni* primers (0.8 µM final) , 0.12 µl of 100 µM *C. coli* primers ( 0.4 µM final), 0.15 µl of 10 µM *C. lari* primers (0.05 µM final), 0.6 µl of each 10 µM probe (0.2 µM final), 7.18 µl of nuclease-free water (Promega, USA) and 5 µl of DNA for standard curve construction.

For unknown samples (food samples), the same volume and concentrations of all components were used except for nuclease-free water (2.18 µl) and DNA (10 µl).

Final volume: 30 µl

**Reaction volume and amount of cDNA/DNA (E)**

Reaction volume: SYBR Green melt curve assay 25 µl; singleplex qPCR assay 25 µl; multiplex qPCR assay 30 µl

DNA volume for standard curves: 5 µl; real copy number of genomes in a well was determined using the formula [[2](#_ENREF_2)]:

Genome copies/µl = (C x N_A_ x 10^-9^)/(genome length (bp) x M_w_)

Where C is a measured concentration of extracted DNA (ng/µl), N_A_ is Avogadro's number (6.02 x 10^23^ molecule/mole) and M_w_ is molecular weight of 1 bp (660 Da).

Genome length (bp):

*C. jejuni* 1641481 (NCBI NC_002163); *C. coli* 1714000 [[4](#_ENREF_4)]; *C. lari* 1525460 (NCBI NC_012039)

DNA volume for unknown samples: 10 µl; quantification in accordance with standard curves

**Primer, probe, Mg^2+^, and dNTP concentrations (E)**

SYBR Green assay: 0.4 µM primers

Singleplex qPCR: 0.4 µM primers and 0.2 µM probe

Optimized multiplex qPCR: 0.8 µM *C. jejuni* primers, 0.4 µM *C. coli* primers, 0.05 µM *C. lari* primers, 0.2 µM probes

Mg^2+^ and dNTPs were components of commercial 2 × TaqMan® Universal PCR Master Mix, No AmpErase® UNG (Applied Biosystems, USA) and 2 × Power SYBR Green PCR Master Mix (Applied Biosystems, USA), concentrations unknown

**Polymerase identity and concentration (E)**

AmpliTaq Gold® DNA Polymerase - a component of commercial 2 × TaqMan® Universal PCR Master Mix, No AmpErase® UNG (Applied Biosystems, USA) and 2 × Power SYBR Green PCR Master Mix (Applied Biosystems, USA), concentrations unknown

**Buffer/Kit identity and manufacturer (E)**

2 × TaqMan® Universal PCR Master Mix, No AmpErase® UNG (Applied Biosystems, USA)

2 × Power SYBR Green PCR Master Mix (Applied Biosystems, USA)

**Additives (SYBR Green I, DMSO, and so forth), (E)**

Not applied

**Manufacturer of plates/tubes and catalogue number (D)**

MicroAmp® Optical 96-Well Reaction Plate (catalogue number 4306737, Applied Biosystems, USA)

**Complete thermocycling parameters (E)**

Melt curve analysis for primers' specificity and secondary structures formation check: from 60°C to 95°C

Singleplex and multiplex qPCR: 95°C for 10 min, followed by 40 cycles consisting of 95°C for 20 s and 60°C for 60 s

**Reaction setup (manual/robotic), (D)**

Manual

**Manufacturer of qPCR (E)**

7500 Real-Time PCR System (Applied Biosystems, USA)

**8. qPCR validation**

**Specificity (gel, sequence, melt or digest), (E)**

Horizontal agarose-gel electrophoresis

Melt curve analysis (from 60°C to 95°C)

**For SYBR Green I, Cq of the NTC (E)**

No signal detected in NTCs, Cq of the NTCs undetermined or higher than 38

**Calibration curves with slope and y intercept (E)**

Singleplex:

*hipO* (slope = -3.757; y-intercept = 41.308); *glyA* (slope = -3.61; y-intercept = 37.393); *pepT* (slope = -3.996; y-intercept = 40.234)

Multiplex with DNA sample from each strain individually:

*hipO* (slope = -3.549; y-intercept = 43.818); *glyA* (slope = -3.443; y-intercept = 41.653); *pepT* (slope = -3.604; y-intercept = 41.966)

Multiplex with mixed DNA sample:

*hipO* (slope = -3.565; y-intercept = 42.227); *glyA* (slope = -3.399; y-intercept = 40.040); *pepT* (slope = -3.506; y-intercept = 39.548)

**PCR efficiency calculated from slope (%), (E)**

Singleplex:

*hipO* (E = 84.56); *glyA* (E = 89.24); *pepT* (E = 77.93)

Multiplex with DNA sample from each strain individually:

*hipO* (E = 91.347); *glyA* (E = 95.229); *pepT* (E = 92.117)

Multiplex with mixed DNA sample:

*hipO* (E = 90.848); *glyA* (E = 96.973); *pepT* (E = 92.888)

**R^2^ of calibration curve (E)**

Singleplex:

*hipO* (R^2^ = 0.999); *glyA* (R^2^ = 1.0); *pepT* (R^2^ = 0.993)

Multiplex with DNA sample from each strain individually:

*hipO* (R^2^ = 0.999); *glyA* (R^2^ = 0.998); *pepT* (R^2^ = 0.998)

Multiplex with mixed DNA sample:

*hipO* (R^2^ = 0.998); *glyA* (R^2^ = 0.998); *pepT* (R^2^ = 0.998)

**Linear dynamic range (E)**

Eight points of ten-fold serial dilutions in the range from approximately 10^0^ to 10^7^ genome copies/well were tested for multiplex qPCR

In the tested range, reactions were linear from 10^1^ to 10^7^ genome copies/well, and had potential to cover wider range at higher orders of magnitude

**Cq variation at LOD (E)**

0.4 – 0.7 cycle

**Evidence for LOD (E)**

No amplification after 38 cycles

**If multiplex, efficiency (%) and LOD of each assay (E)**

Multiplex with DNA sample from each strain individually:

*hipO* (E = 91.347); *glyA* (E = 95.229); *pepT* (E = 92.117)

Multiplex with mixed DNA sample:

*hipO* (E = 90.848); *glyA* (E = 96.973); *pepT* (E = 92.888)

(this section above – PCR efficiency calculated from slope)

LOD (CFU equivalent/well) for optimized multiplex qPCR with mixed DNA:

Cut off cycle 38 at 95% confidence level

*C. jejuni:* 6.62 < LOD < 16.10

*C. coli:* 5.13 < LOD < 6.30

*C. lari:* 4.87 < LOD < 5.23

**9. Data analysis**

Six independent experiments were conducted and data analysed

**qPCR analysis program (source, version), (E)**

Instrument compliant 7500 Software (Applied Biosystems, USA, version 2.0.5)

Microsoft Office Excel 2007 (Microsoft, USA, version 2007)

GenEx software (MultiD Analyses AB, Sweden, version GenEx 5 Enterprise)

**Method of Cq determination (E)**

Second derivative maximum

**Outlier identification and disposition (E)**

Grubbs test (MultiD Analyses AB, Sweden, version GenEx 5 Enterprise)

**Results for NTCs (E)**

No signal detected in NTCs

Cq of the NTCs undetermined or higher than 38

**Justification of number and choice of reference genes (E)**

Not applied

**Description of normalization method (E)**

Not applied

**Number and stage (reverse transcription or qPCR) of technical replicates (E)**

All samples were run in duplicates; non-template (NTC) and positive controls were included

**Repeatability (intraassay variation), (E)**

Cq deviation from mean Cq expressed as SD

*C. jejuni* – singleplex SD mean 0.039; optimized multiplex SD mean 0.118

*C. coli* – singleplex SD mean 0.318; optimized multiplex SD mean 0.104

*C. lari* – singleplex SD mean 0.091; optimized multiplex SD mean 0.091

**Statistical methods for results significance (E)**

Not applied

**Software (source, version), (E)**

# GenEx software (MultiD Analyses AB, Sweden, version GenEx 5 Enterprise)

Microsoft Office Excel 2007 (Microsoft, USA, version 2007)

References

1. Kalendar R, Lee D, Schulman AH: **FastPCR software for PCR primer and probe design and repeat search.** *Genes, Genomes and Genomics* 2009, **3:**1-14. [www.biocenter.helsinki.fi/bi/programs/fastpcr.htm]

2. He YP, Yao XM, Gunther NW, Xie YP, Tu SI, Shi XM: **Simultaneous detection and differentiation of *Campylobacter jejuni*, *C. coli*, and *C. lari* in chickens using a multiplex real-time PCR assay.** *Food Anal Methods* 2010, **3:**321-329.

3. LaGier MJ, Joseph LA, Passaretti TV, Musser KA, Cirino NA: **A real-time multiplexed PCR assay for rapid detection and differentiation of *Campylobacter jejuni* and *Campylobacter coli*.** *Mol Cell Probes* 2004, **18:**275-282.

4. Chang N, Taylor DE: **Use of pulsed-field agarose-gel electrophoresis to size genomes of *Campylobacter* species and to construct a *Sal*I map of *Campylobacter jejuni* UA580.** *J Bacteriol* 1990, **172:**5211-5217.
